# 6-hydroxydopamine and ovariectomy has no effect on heart rate variability parameters of females

**DOI:** 10.6061/clinics/2021/e3175

**Published:** 2021-09-28

**Authors:** Tomás de la Rosa, Viviam Sanabria Calvo, Valeria Cassia Gonçalves, Débora Amado Scerni, Fúlvio Alexandre Scorza

**Affiliations:** Departamento de Neurologia, Escola Paulista de Medicina/Universidade Federal de Sao Paulo (EPM/UNIFESP), Sao Paulo, SP, BR.

**Keywords:** 6-OHDA, Female, Ovariectomy, HRV

## Abstract

**OBJECTIVES:**

In addition to the classic motor symptoms of Parkinson’s disease (PD), patients also present with non-motor symptoms, such as autonomic dysfunction, which is present in almost 90% of patients with PD, affecting the quality of life and mortality. Regarding sex differences in prevalence and presentation, there is increasing concern about how sex affects autonomic dysfunction. However, there are no previous data on autonomic cardiac function in females after 6-hydroxydopamine (6-OHDA) striatal injection.

**METHODS:**

Wistar female rats were ovariectomized. After 20 days, the animals received bilateral injections of 6-OHDA (total dose per animal: 48 µg) or a vehicle solution in the striatum. Thirty days after 6-OHDA injection, subcutaneous electrodes were implanted for electrocardiogram (ECG) recording. Ten days after electrode implantation, ECG signals were recorded. Analyses of heart rate variability (HRV) parameters were performed, and the 6-OHDA lesion was confirmed by analyzing the number of tyrosine hydroxylase-positive neurons in the substantia nigra pars compacta (SNpc).

**RESULTS:**

A high dose of 6-OHDA did not affect HRV of females, independent of ovariectomy. As expected, ovariectomy did not affect HRV or lesions in the SNpc after 6-OHDA injection.

**CONCLUSIONS:**

We suggest that females with 6-OHDA present with cardioprotection, independent of ovarian hormones, which could be related to female vagal predominance.

## INTRODUCTION

Parkinson’s disease (PD) is a neurodegenerative disease that affects 1% of the general population and 3% of the population over 60 years of age (1). This prevalence is expected to double in the next few decades due to demographic aging (2). PD is classically characterized by motor clinical manifestations caused by the loss of dopaminergic neurons in the nigrostriatal pathway. However, PD patients also present with non-motor symptoms that could precede motor dysfunction by up to 10 years (3). Non-motor symptoms are related to autonomic function, which affects up to 90% of patients (4). Autonomic dysfunction in patients with PD has a great impact on their quality of life; specifically, cardiac autonomic dysfunction also increases mortality risk, which is two times higher in PD patients than that in the general population (5). The most common causes of death among patients with PD are of cardiovascular and respiratory origin (5), and as previously reported in the literature, this event can occur suddenly and unexpectedly (6-8).

An animal model widely used for PD research is induced by the injection of 6-hydroxydopamine (6-OHDA) into the striatum (CPu) or directly into the substantia nigra pars compacta (SNpc) in rodents, causing a specific retrograde lesion of catecholaminergic neurons due to the increase in reactive oxygen species (9). Striatal 6-OHDA also causes neuronal loss in central regions related to autonomic cardiovascular control, such as the dorsal motor nucleus of the vagus (10), nucleus of the solitary tract, and nucleus ambiguus (11). After bilateral injection of 6-OHDA, male rodents showed decreased heart rate variability (HRV) (12), bradycardia, and mean arterial pressure reduction (13,14). Although these autonomic dysfunctions were assessed after both low and high doses of 6-OHDA, a high impairment was observed after a high dose and long after -injection period. This is due to the 6-OHDA infusion mechanism through the nigrostriatal pathway to the medullary regions involved in cardiovascular control, which requires higher doses than models to assess nigrostriatal dopaminergic loss. Studies using the same dose of 6-OHDA have proposed the periaqueductal gray matter as the connection between the nigrostriatal pathway and medullary regions (15). Therefore, a high dose injection of 6-OHDA in rodents is an adequate model to study cardiac autonomic dysfunction in PD.

Epidemiological studies have widely reported a higher prevalence of PD in males than in females (3); this difference is more pronounced in the early stages of the disease (16). Sex differences in the prevalence and presentation of PD have been associated with the neuroprotective potential of sex hormones, such as estrogen (17). After 6-OHDA injection, the females had a preserved nigrostriatal pathway compared to males and ovariectomized females (18,19). However, this neuroprotective phenomenon was only observed using small doses of 6-OHDA (1−5 µg) and after injection times of 1−2 weeks. With a high dose of 6-OHDA or long after injection time, these effects were no longer replicated (20). The authors of this study named this phenomenon as the “critical period for nigrostriatal neuroprotection,” suggesting that in the 6-OHDA model, the physiological neuroprotection provided by sex hormones is time-dependent, both under physiological and supplemented conditions.

Some studies have already explored the participation of sex hormones in cardiovascular and autonomic function in PD, using the 6-OHDA model and ovariectomized rats with hormonal treatment (21). However, to date, this is the first study to use a high dose of 6-OHDA, which, according to the literature, causes a high autonomic impairment in males. This, in addition to the new policies in animal research that involve sex (22), justifies the need for this kind of experimental approach.

## MATERIALS AND METHODS

### Animals

The experiments were conducted on 25 female Wistar rats weighing 250−320g and aged 8 weeks. The animals were kept in cages with four individuals with appropriate sawdust and free access to food and water in a light-dark cycle (12/12h) at a temperature of 21±2 °C. Animals were purchased from the Central Bioterium of the Federal University of São Paulo and were kept in the Bioterium of the Neuroscience Laboratory. The procedures were performed according to the guidelines of the Ethics Committee of the Federal University of São Paulo (Protocol number: 7456260617), and every effort was made to minimize animal suffering. The animals were divided into four experimental groups:

Sham: non-ovariectomized females that received saline injections

Sham + OVX: ovariectomized females that received saline injections

6-OHDA: non-ovariectomized females that received 6-OHDA injections

6-OHDA + OVX: ovariectomized females that received 6-OHDA injections

### Vaginal Cytology and Ovariectomy Procedure

The estrous cycle of female rats was followed for at least two complete cycles (8−10 days) before performing any experimental procedure. The estrous cycle was assessed by vaginal cytology, and the cellular composition of the vaginal smears was investigated under a microscope. Only rats with a regular estrous cycle were included in the study.

For ovariectomy, anesthesia was induced with ketamine (90 mg/kg i.p.) and xylazine hydrochloride (10 mg/kg i.p.). Anesthesia was assessed by the ocular and paw reflexes, the lateral regions of the hair were removed, two incisions of 1 cm on each side were made, and the ovaries were pinched and removed. After surgery, analgesic tramadol (20 mg/kg i.p.), anti-inflammatory meloxicam (1 mg/kg s.c.), and antibiotic enrofloxacin (5 mg/kg i.p.) were administered. This procedure was performed in the OVX groups, while in the other groups, ovaries were kept intact, without performing any sham surgery, and all the following procedures were performed in the estrous physiological phase of the cycle.

### Stereotaxic Surgery

Twenty days after ovariectomy, the animals were anesthetized with ketamine (90 mg/kg i.p.) and xylazine hydrochloride (10 mg/kg i.p.), anesthetized with ocular and paw reflexes, and placed in a stereotactic device (EFF 331-Insight^TM^). A 10 µL Hamilton syringe attached to the stereotactic rod was used to inject 1 µL of 6-OHDA (H4381, Sigma^©^, Missouri, USA) solution (12 µg/µL concentration in 0.3% ascorbic acid) or a saline solution bilaterally in four different coordinates in the CPu: (1) laterolateral: -2.7 mm, anteroposterior: bregma, dorsoventral: -4.5 mm; (2) laterolateral: -3.2 mm, anteroposterior: +0.5 mm, dorsoventral: -4.5 mm; (3) laterolateral: +2.7 mm, anteroposterior: bregma, dorsoventral: -4.5 mm; and (4) laterolateral: +3.2 mm, anteroposterior: +0.5 mm, dorsoventral: -4.5 mm. The coordinates and dose followed the anatomic atlas guidelines (23) and previous studies (24,25).

### Implantation of Cardiac Electrodes and Electrocardiogram (ECG) Recording

Thirty days after the injection of 6-OHDA or saline into the CPu, anesthesia was induced with ketamine (90 mg/kg i.p.) and xylazine hydrochloride (10 mg/kg i.p.) and assessed by the ocular and paw reflexes; the animals were then placed in a stereotactic device (EFF 331-Insight^TM^, São Paulo, Brazil). The electrode output connector was positioned above the skullcap of the animal using a self-curing acrylic resin. The stainless steel coated with nylon electrode wires was subcutaneously guided to the xiphoid process and the central region of the sternocleidomastoid muscles.

After recovery from the surgery (7 days), ECGs of resting non-anesthetized animals were recorded for 5 days before euthanasia. The ECG was recorded and transduced by an amplification circuit and then digitized using a PowerLab v8 device (ADInstruments) at a sampling frequency of 1000 Hz and transferred to LabChart v8 software (ADInstruments, Australia) for processing. The ECG analysis and HRV parameters were evaluated using the LabChart tools available for this purpose. For the analysis, we chose the last register from 5 days in the case of the OVX animals and the register in which animals were in the estrous phase in the case of intact animals. The first 30 minutes of the signal was discarded, and four stable periods of 5 minutes were selected for analysis. The following parameters were calculated: heart rate (HR), the mean interval between adjacent R waves (RRi), the corrected interval between Q and T waves (QTc), the standard deviation of all normal-to-normal intervals (SDNN), the square root of the mean of the squared differences between adjacent normal RRi (RMSSD), the low-frequency component in normalized units (LF), the high-frequency component in normalized units (HF), and the ratio between LF and HF (LF/HF).

### Immunohistochemistry

After collecting the functional data, the animals were euthanized using the transcardiac perfusion method after the intraperitoneal injection of sodium thiopental (80 mg/kg). The rib cage was opened by introducing a needle into the left ventricle for the injection of 150 ml of phosphate-buffered saline (PBS; 0.1 M; pH 7.4) and 4% paraformaldehyde (PFA; 0.1 M; pH 7.4). An incision in the right atrium was made to facilitate the draining of the solution. The brain was removed and placed in 4% PFA for 24h and then in a 30% cryoprotective sucrose solution for 72h to further cut it into 40 µm coronal sections using a cryostat (Microm HM 505E). Five sections of the SNpc were selected for staining, separated by 240 µm starting from the bregma -4.56 (23). Tyrosine hydroxylase (TH) was detected using mouse anti-TH (ab76442, Abcam^®^, dilution 1:1000) as the primary antibody, diluted in PBS containing 1% normal donkey serum and 0.3% Triton X-100 and incubated for 48h. Subsequently, the slices were washed with PBS before being incubated with a biotinylated secondary antibody for 24h (ab6720, Abcam^®^, dilution 1:1500). Slices were incubated with avidin-biotin-peroxidase (VECTASTAIN^®^, California, USA). The sections were then stained with 3,3’-diaminobenzidine tetrachloride dissolved in 0.05 M Tris-HCl (pH 7.6) and further activated with 0.3% hydrogen peroxide. The sections were rinsed again in PBS and mounted sequentially in a rostrocaudal order onto slides on gelatin-coated slides, dehydrated through a series of ethanol concentrations and xylene, and finally mounted with Dibutylphthalate Polystyrene Xylene for mounting. Photomicrographs were taken using an optical microscope (Nikon, ECLIPSE E600) and ImageJ for cell counting analysis of TH^+^-stained neurons.

### Statistical Analysis

Data were initially tested for normality distribution using the Shapiro-Wilk test, which confirmed that the HRV data had a normal distribution, while the data of the immunochemistry of the SNpc did not. Based on these results, we performed a general linear model multivariate for the HRV data followed by the Sidak post hoc test and a nonparametric Kruskal-Wallis test for the immunohistochemistry data followed by the Dunn-Bonferroni post hoc test. Levene’s test was also performed to confirm that all data had homogeneity of variance, and a significance of α<0.05 was adopted for all tests. Considering a minimal power (β) of 80%, the sensitivity threshold of the present study detected effect sizes larger than Cohen’s f=0.97 or Eta squared (η^2^)=0.486.

Statistical analysis was performed using SPSS Statistics 21.0 (IBM^®^ SPSS Products: Statistics Common). Graphics were built using GraphPad Prism 6.01 (GraphPad Software. Inc^®^). Effect size calculations were performed using GPower 3.1.

## RESULTS

### TH Immunohistochemistry

**Figure 1:**
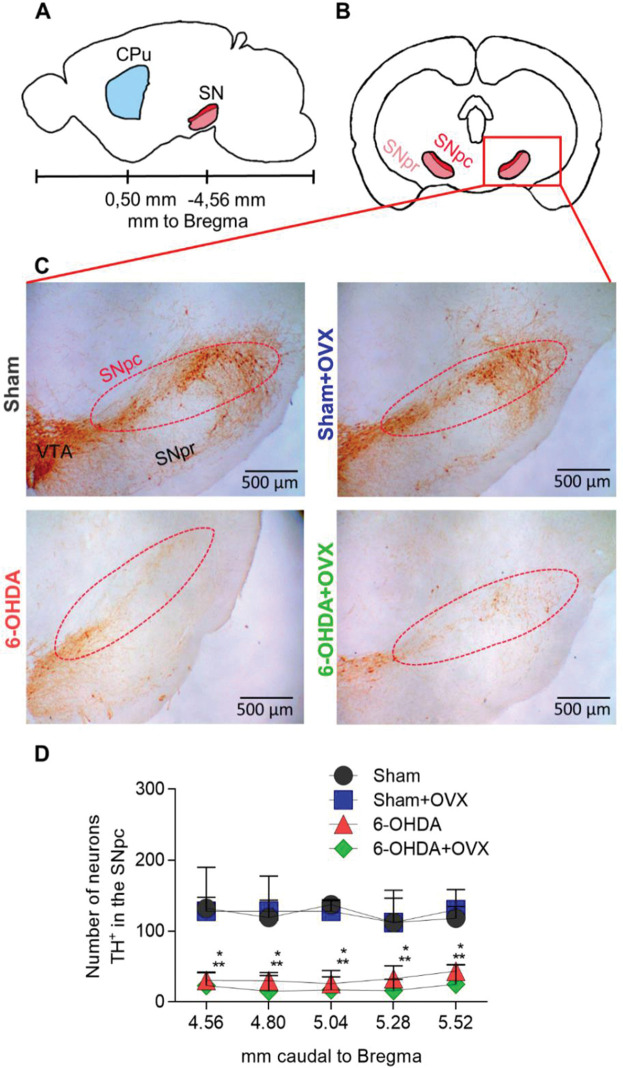
Number of TH+ neurons in the SNpc 40 days after striatal injection of 6-OHDA **(A)** Position of the CPu and SNpc in relation to the Bregma. **(B)** Coronal view of the SNpc and **(C)** photomicrographs of the SNpc neurons TH^+^ stained. **(D)** The number of TH+ neurons in the SNpc represented as Median±interquartile range. Data are analyzed using the Kruskal-Wallis test and Dunn-Bonferroni post-hoc test. *p<0.001 compared to Sham **p<0.001 compared to Sham+OVX. Sham, n=9; Sham+OVX, n=5; 6-OHDA, n=6; 6-OHDA+OVX, n=5. (SNpc, substantia nigra pars compacta; SNpr, substantia nigra pars reticulata; VTA, ventral tegmental area; TH, tyrosine hydroxylase; 6-OHDA, 6-hydroxydopamine; OVX, ovariectomized).

To analyze the effect of the injection of 6-OHDA or saline into the CPu, the number of TH+ neurons in the SNpc was analyzed (Figures 1A-B). The analysis of the number of TH^+^ neurons in the SNpc showed a significant reduction (*p*<0.001) in animals that received 6-OHDA injection (mean values from the five sections analyzed: Sham, 119.4±1.33 TH+ cells; Sham+OVX, 129.4±45.9; 6-OHDA, 30.5±18.4; and 6-OHDA+OVX, 19.8±23.2) (Figures 1C-D). This reduction was observed in all five levels of the SNpc analyzed. Ovariectomy had no effect on the number of TH^+^ neurons in the SNpc in any of the five levels analyzed, and the analysis did not show an interaction between ovariectomy and 6-OHDA injection.

### Heart Rate Variability

**Figure 2:**
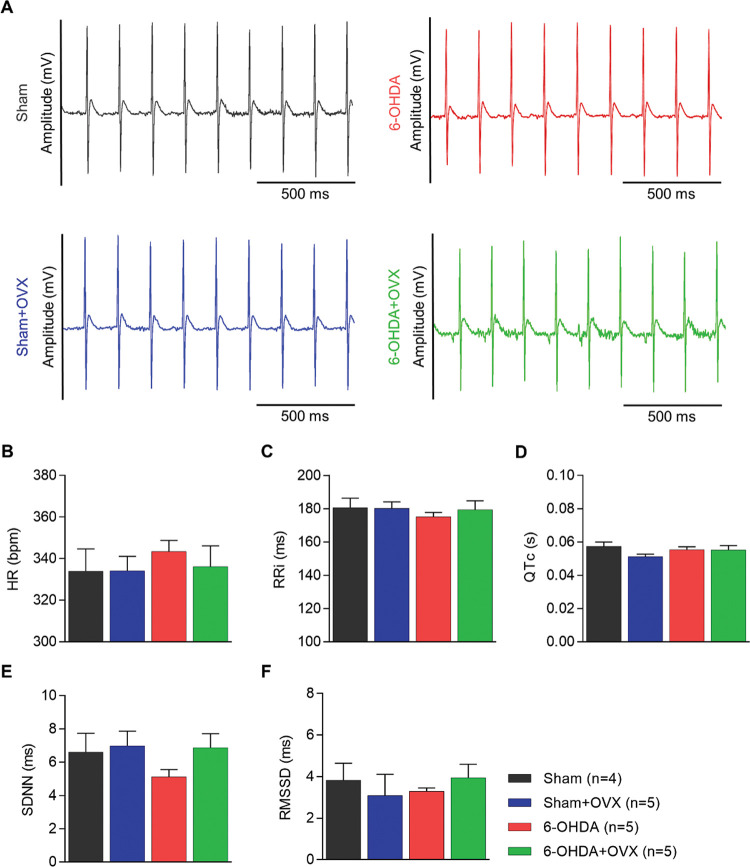
Heart rate variability parameters (time domain analysis) **(A)** Electrocardiographic signal for each group. **(B)** Heart rate (HR), **(C)** the R-R interval (RRi), **(D)** the Q and T interval (QTc), **(E)** the standard deviation of all normal-to-normal intervals (SDNN), and **(F)** the square root of the mean of the squared differences between adjacent normal RRi (RMSSD). Data are expressed as mean±standard error of the mean. Data are analyzed using GLM multivariate with the Sidak post-hoc test. Sham, n=4; Sham+OVX, n=5; 6-OHDA, n=5; 6-OHDA+OVX, n=5 (6-OHDA, 6-hydroxydopamine; OVX, ovariectomized).

HRV parameters were analyzed using ECG signals (Figure 2A). This analysis did not show a significant effect of 6-OHDA (F_1,19_=0.479; *p*=0.5; η^2^=0.031) or ovariectomy (F_1,19_=0.177; *p*=0.68; η^2^=0.012) on HR (Figure 2B). The values of all groups were approximately 320−340 bpm. The RRi (Figure 2C) and QTc (Figure 2D) did not show any significant effects on the independent variables. The SDNN (6-OHDA, F_1,19_=0.869, *p*=0.36, η^2^=0.055; OVX, F_1,19_=0.671, *p*=0.193, η^2^=0.110) (Figure 2E) and RMSSD (6-OHDA, F_1,19_=0.436, *p*=0.52, η^2^=0.03; OVX, F_1,19_=0.172, *p*=0.685, η^2^=0.012) (Figure 2F) were statistically equivalent in all four groups, suggesting no global effect in the time domain analysis.

**Figure 3:**
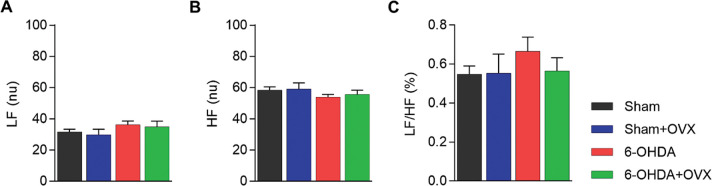
Frequency domain analysis of heart rate variability **(A)** LF. **(B)** HF. **(C)** LF/HF. Data are expressed as mean ± standard error of the mean. Data are analyzed using GLM multivariate with the Sidak post-hoc test. (LF, the low-frequency component in normalized units; HF, the high-frequency component in normalized units; LF/HF, the ratio between LF and HF; 6-OHDA, 6-hydroxydopamine; OVX, ovariectomized).

The frequency domain analysis of HRV did not show an effect of 6-OHDA or ovariectomy. The LF (6-OHDA, F_1,19_=2.125, *p*=0.167, η^2^=0.132; OVX, F_1,19_=0.233, *p*=0.637, η^2^=0.016) and HF (6-OHDA, F_1,19_=1.778, *p*=0.204, η^2^=0.113; OVX, F_1,19_=0.157, *p*=0.698, η^2^=0.011) were approximately 30 and 60 normalized units, respectively (Figures 3A-B), and the ration between them (Figure 3C) was approximately 0.5−0.7 in all experimental groups.

### Tachogram Distribution Analysis

**Figure 4:**
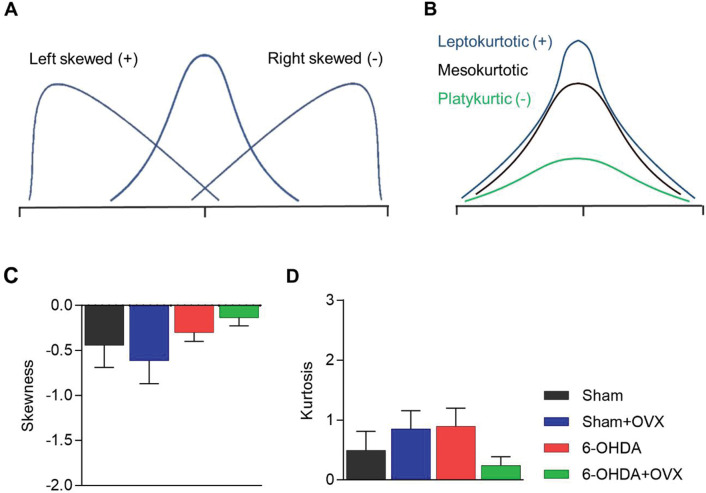
RRi distribution analysis **(A)** Representation of the skewness measure in a distribution. **(B)** Representation of kurtosis in a distribution. **(C)** Skewness. **(D)** Kurtosis. Data are expressed as mean ± standard error of the mean. Data are analyzed using GLM multivariate with the Sidak post-hoc test (6-OHDA, 6-hydroxydopamine; OVX, ovariectomized).

The RRi tachogram was plotted in a histogram, from which we calculated the skewness and kurtosis parameters. The skewness and kurtosis of the distribution provide an alternative measure of the RRi distribution, where the tachogram is analyzed using a non-linear approach. Skewness would inform us about the position of the peak of the distribution compared to its central parameters (Figure 4A), while kurtosis provides a metric of how data are distributed around the central parameter (Figure 4B). The results of this analysis showed a negative skewness in all groups; however, there was no statistically significant difference between the groups (6-OHDA, F_1,18_=2.606, *p*=0.129, η^2^=0.157; OVX, F_1,18_=0.001, *p*=0.988, η^2^=0.001) (Figure 4C). All groups showed a relatively mesokurtic distribution, with no differences between the groups (6-OHDA, F_1,18_=0.151, *p*=0.704, η^2^=0.011; OVX, F_1,18_=0.288, *p*=0.6, η^2^=0.02) (Figure 4D).

## DISCUSSION

PD etiology is characterized by its heterogeneity, which is observed in clinical practice, and morbidity and mortality of patients. This plurality of clinical phenomena have its parallel in the study of pathological processes, where most of the mechanisms and causal agents of the disease are still unknown. Empirical data obtained in preclinical experiments with animal models play a crucial role in unveiling these mechanisms and causes. Experimental approaches, despite controlling and homogenizing the conditions of the experiments, should also consider the heterogeneity present in pathological processes. In the present study, we studied, a phenomenon sometimes forgotten, cardiac function in PD, especially in the population of female rodents.

In the last 20 years, the neuroprotective role of estrogen in the nigrostriatal pathway has been widely studied, showing that low doses of 6-OHDA in rodents cause different effects in males, females, and ovariectomized females (18,26). However, it has also been observed that such neuroprotection is time-and dose-dependent, disappearing when the lesion in the SNpc is above 60% of the dopaminergic neurons (20,27). In our study, we used a high dose of 6-OHDA (48 µg) and a high establishment time of the model (40 days), resulting in a significant reduction in TH^+^ neurons in the SNpc of animals that received 6-OHDA injection compared to the sham animals (Figure 1D). This reduction was approximately 2/3 of TH^+^ neurons in the SNpc, corresponding to 60-75% of the total population. As previously reported in the literature, animals that received 6-OHDA with this size of the lesion were not expected to show any effect of ovariectomy on the TH^+^ neuron population. We did not observe any effect of ovariectomy on SNpc lesions (Figure 1D), contributing to the hypothesis that there is no neuroprotective effect at high doses and after injection.

Autonomic cardiac function is altered in PD, as observed through indirect measures of HRV in patients (28-30), and this reduction has been associated with an increased risk of mortality (28,31). Similar results were observed using the 6-OHDA model in males (12), where HRV decreased compared to controls. However, in some animal studies using low doses, this reduction in HRV has not been observed in males (32) and females (21). The present study is the first to test the effect of a high dose of 6-OHDA in females, and we did not observe any effect in the time (Figure 2) or frequency domain analyses (Figure 3). In a previous study in which HRV was reduced after 6-OHDA injection in males, Gonçalves et al. (12) reported differences between groups in HR (Cohen’s f=2.67), the SDNN (Cohen’s f=1.75), and the RMSSD (Cohen’s f=1.13). The effect sizes observed in this study were all higher than the threshold of detection in the present study (Cohen’s f=0.97) (refer to Methods, Statistical Analysis). This suggests that the same dose of 6-OHDA and the establishment time elicit HRV reductions in males but not in females. We suggest that this difference may be due to some cardioprotective mechanism, which in either way does not depend on ovarian hormones, as ovariectomy did not influence HRV parameters. However, other mechanisms, such as hormone production by other tissues or interaction with other hormonal axes cannot be discarded; this is a limitation of the ovariectomy method for studying hormonal interactions.

Compared with males, females have a higher vagal tone; however, this difference appears to wear off after menopause (33). Estrogen increases vagal tone in male and female rodents (34), and ovariectomy appears to increase the sympathetic activity after the surgery (35), but this effect was no longer observed 14 (21) and 21 (36,37) days after ovariectomy. These differences in autonomic function response to ovariectomy could be explained not only as an adaptation mechanism of the system to new hormonal levels but also as an effect of steroidal hormone production by other tissues, such as the adrenal glands, the adipose tissue, or even the brain. In both clinical (28,30) and animal (12) studies, HRV reduction was especially present in parameters reflecting vagal function, such as the RMSSD. Furthermore, in males, a reduction in choline acetyltransferase-positive neurons in the nucleus ambiguus after 6-OHDA injection (11) was observed, in addition to neuronal loss in the nucleus of the solitary tract, a region responsible for afference of peripheral chemo and mechanoreception. Altogether, this functional and anatomical evidence indicates that cardiac autonomic dysfunction observed through HRV reduction could involve decreased vagal function in males, and this has not been replicated in females, suggesting some cardioprotective mechanism. Further investigation is needed to elucidate whether functional cardioprotection in females correlates with conserved anatomical structures.

To further explore this hypothesis, we performed a non-linear analysis of the tachogram distribution. We did not observe any differences in the distribution shapes between the groups (Figure 4). However, we found that females had negative right-skewed distributions, which correlated with high RRi values. This contrasts with previous results in males using the same non-linear analysis, where a positive left-skewed distribution was observed (12). The skewness of the distribution could be interpreted as a tendency toward low or high values of RRi, indicating a predominance of sympathetic or vagal control, respectively. In line with this, our results agree with the previous literature that indicates a vagal predominance in females (34,35), although, in the time domain analysis, the vagal parameters did not show differences with male studies. The different calculation methods and the linear versus non-linear nature of both analyses could account for these differences. Regarding kurtosis, males showed higher values after 6-OHDA injection (12) than females, indicating a leptokurtic distribution (Figure 4). Kurtosis has previously been interpreted as a metric of cardiac rigidity (12,38), suggesting a more rigid cardiac function in males than in females. However, the nature of this analysis makes it extremely sensitive to changes between the methodologies used for the HRV analysis, especially the sample size of the tachogram. Considering this, it would be wise to use it as an internal comparison metric, avoiding comparisons between studies.

In this study, our results showed that, in contrast to males, a high dose of 6-OHDA did not affect the autonomic cardiac function of females. However, the study has some limitations that need to be stated, explaining how we have overcome them. First, the sample size for the HRV analysis was small, which could be a risk factor for a type II error. In this regard, the effect size calculation has been presented together with each test performed and in every comparison with previous literature. This is not only the best tool against a type II error but also the only one possible to properly and carefully communicate our findings. Second, there is a limitation in the use of ovariectomy as a procedure to test the role of sex hormones in normal and pathological physiology. It is widely known and has been previously mentioned here that after ovariectomy, other tissues continue to synthesize these hormonal compounds. Approaches with aromatase inhibitors may provide further insights into this question. Finally, as mentioned previously, the diversity of HRV analysis tools and methods available empowers the exploration of cardiac electrical activity in many ways, depending on our experimental design (39). Despite the benefits of these technologies, it is sometimes difficult to standardize methods and analyses, hindering the comparison of results among different studies.

## CONCLUSION

In summary, this is the first study to evaluate autonomic cardiac function in ovariectomized and intact females receiving a high dose of 6-OHDA striatal injection. Due to the high dose and establishment time, no effect of ovariectomy on the number of dopaminergic neurons in the SNpc was observed. Additionally, we did not observe any changes in HRV of females in any of the parameters analyzed. This contrasts with previous evidence suggesting autonomic impairment in males who received 6-OHDA striatal injections. We suggest an increased vagal tone in females as a possible cardioprotective mechanism. Future studies that evaluate cardiac autonomic control in PD may elucidate the mechanisms underlying these sex differences.

## AUTHOR CONTRIBUTIONS

de la Rosa T was responsible for the study conceptualization, methodology, investigation, formal analysis, data curation, manuscript original drafting, review and editing. Calvo VS was responsible for the study methodology, investigation, formal analysis, manuscript original drafting, review and editing. Gonçalves VC was responsible for the study investigation, formal analysis, data curation, manuscript original drafting, review and editing. Scerni DA and Scorza FA were responsible for the study conceptualization, methodology, investigation, formal analysis, manuscript original drafting, review and editing.
